# Mechanical and Gamma Ray Absorption Behavior of PbO-WO_3_-Na_2_O-MgO-B_2_O_3_ Glasses in the Low Energy Range

**DOI:** 10.3390/ma14133466

**Published:** 2021-06-22

**Authors:** Aljawhara H. Almuqrin, Badriah Albarzan, O. I. Olarinoye, Ashok Kumar, Norah Alwadai, M. I. Sayyed

**Affiliations:** 1 Department of Physics, College of Science, Princess Nourah bint Abdulrahman University, Riyadh 11671, Saudi Arabia; ahalmoqren@pnu.edu.sa (A.H.A.); Baalbarzan@pnu.edu.sa (B.A.); nmalwadai@pnu.edu.sa (N.A.); 2 Department of Physics, Federal University of Technology, Minna 920271, Nigeria; leke.olarinoye@futminna.edu.ng; 3 Department of Physics, University College, Benra-Dhuri, Punjab 148024, India; ajindal9999@gmail.com; 4 Department of Physics, Punjabi University, Patiala, Punjab 147002, India; 5 Department of Nuclear Medicine Research, Institute for Research and Medical Consultations (IRMC), Imam Abdulrahman bin Faisal University (IAU), P.O. Box 1982, Dammam 31441, Saudi Arabia

**Keywords:** Makishima and Mackenzie model, glasses, Young’s modulus, low energy, shielding

## Abstract

The Makishima and Mackenzie model has been used to determine the mechanical properties of the PbO-WO_3_-Na_2_O-MgO-B_2_O_3_ glass system. The number of bonds per unit volume of the glasses (n_b_) increases from 9.40 × 10^22^ to 10.09 × 10^22^ cm^−3^ as the PbO content increases from 30 to 50 mol%. The Poisson’s ratio (σ) for the examined glasses falls between 0.174 and 0.210. The value of the fractal bond connectivity (d) for the present glasses ranges from 3.08 to 3.59. Gamma photon and fast neutron shielding parameters were evaluated via Phy-X/PSD, while that of electrons were calculated via the ESTAR platform. Analysis of the parameters showed that both photon and electron attenuation ability improve with the PbO content. The fast neutron removal cross section of the glasses varies from 0.094–0.102 cm^−1^ as PbO molar content reduced from 50–30 mol%. Further analysis of shielding parameters of the investigated glass system showed that they possess good potential to function in radiation protection applications.

## 1. Introduction

Nuclear medicine utilizes different radiopharmaceuticals for treatment of a number of diseases. One of the recent techniques applied widely for diagnostic molecular imaging is the positron emission tomography (PET) and computer tomography (CT). Other techniques are also used, which depend mainly on the use of a specific type of radiation in different medical fields for the purposes of radiation treatments [1]. As a result of the frequent and continuous use of such devices, workers in medical facilities are exposed to direct exposure to ionizing radiation, and this becomes a real threat to the safety of the workers [2]. It is well known that this direct exposure to radiation is not limited only to workers in medical facilities, but also to the patients who undergo medical diagnosis in nuclear medicine. Thus, one of the most important challenges facing medical workers is to reduce the exposure to radiation, and this is done through a set of preventive procedures. One of the most popular preventive procedures is to take the time and time factors into account. In brief, it can be said that reducing the time of exposure to the radiation as well as increasing the distance between the radioactive source and the workers lead to reduce the unwanted effects of radiation well. Sometimes these two factors cannot be well controlled, and therefore it becomes necessary to use certain materials that have the ability to absorb the radiation and thus mitigate the negative effects of radiation. Such materials, which are widely used in nuclear and radiological fields, are called radiation shielding materials [3,4,5,6,7]. Historically, lead bricks, heavy concretes, alloys and ceramics are common materials used for the shielding purposes in several facilities [8,9,10,11]. Recently, the necessity has become urgent to replace these materials with new transparent materials, and to produce protective eyeglasses against radiation, as well as in the manufacture of window glasses used in the hospital and medical radiology centers [12,13,14].

During the past years to the present day, researchers have developed various types of borate, tellurite, silicate glasses and other common types of glass as promising alternative materials for radiation protection. Usually the radiation protection properties of any new glass system are evaluated through three main methods, including experimental or theoretical studies in addition to the works that are based on the simulation programs [15,16,17,18,19]. Although researchers focus on developing different types of glass as a protective material for radiation, there are few previous studies that have focused on studying the properties of radiation protection at low energies and thus studying the possibility of using the glasses as a radiation protective material in medical facilities, especially those use low energies [20,21,22]. On the other hand, among different glass systems, borate glasses are of interest fundamentally for several reasons such as: they have low melting temperatures, their wide glass-forming range, their compatibility with transition metals and their ability to form glass with heavy metal oxides [23,24]. For borate-based glass systems, heavy metal oxides such as PbO addition can conveniently enhance the density of the glasses to improve the radiation protection efficiencies. The aim of this work is to report the mechanical properties and radiation attenuation factors for PbO-WO_3_-Na_2_O-MgO-B_2_O_3_ glass systems at low energy region (between 22 and 364 keV). 

## 2. Materials and Methods

Previous findings [25] provide all the information necessary for preparing the samples. Table 1 presents the chemical composition of the samples.

The glasses have been coded as:

PbB30: 30 PbO–10 WO_3_–10 Na_2_O–10 MgO–40 B_2_O_3_ (ρ = 4.549 g/cm^3^) 

PbB35: 35 PbO–10 WO_3_–10 Na_2_O–10 MgO–35 B_2_O_3_ (ρ = 4.810 g/cm^3^)

PbB40: 40 PbO–10 WO_3_–10 Na_2_O–10 MgO–30 B_2_O_3_ (ρ = 5.061 g/cm^3^)

PbB45: 45 PbO–10 WO_3_–10 Na_2_O–10 MgO–25 B_2_O_3_ (ρ = 5.149 g/cm^3^)

PbB50: 50 PbO–10 WO_3_–10 Na_2_O–10 MgO–20 B_2_O_3_ (ρ = 5.326 g/cm^3^)

### 2.1. Mechanical Properties

The average cross-link density ($\bar{n_{c}}$) is define by the formula [26]:

(1) $$\bar{n_{c}}=\frac{\sum x_{i}{\left(n_{c}\right)}_{i}{\left(N_{c}\right)}_{i}}{\sum x_{i}{\left(N_{c}\right)}_{i}}$$

where (n_c_ = n_f_ – 2) and n_f_ is the coordination numbers, x_i_ is the mole fraction and N_c_ is the number of cations present in the respective constituents of the glasses. 

The n_b_ is given by [27]:

(2) $$n_{b}=\frac{N_{A}}{V_{m}}\sum {\left(n_{f}\right)}_{i}x_{i}$$

where N_A_ and V_m_ are the Avagadro’s number and the molar volume of the glass, respectively.

According to Makishima and Mackenzie (MM) model, the Young’s modulus (E), bulk modulus (K), shear modulus (G), longitudinal modulus (L), Poisson’s ratio (σ), fractal bond connectivity (d) and hardness (H) are calculated via the next equations [28,29]:

(3) $$E=8.36V_{t}G_{t}$$

(4) $$K=10V_{t}^{2}G_{t}$$

(5) $$G=\frac{30V_{t}^{2}G_{t}}{\left(10.2V_{t}-1\right)}$$

(6) $$L=K+\left(\frac{4}{3}\right)G$$

(7) $$\sigma =0.5-\left(\frac{1}{7.2V_{t}}\right)$$

(8) $$d=4\left(\frac{G}{K}\right)$$

(9) $$H=\frac{\left(1-2\sigma \right)E}{6\left(1+\sigma \right)}$$

where V_t_ and G_t_ are the ionic packing ratio and the dissociation energy per unit volume of the glass, respectively.

### 2.2. Radiation Shielding Parameters and Computation

The attenuation of the radiation is usually described by several parameters depending on the type of radiation and sometimes its energy. For photons, the transmission through an attenuating barrier (of mass thickness $x_{m}$) in a broad beam approximation is easily described by the modified Beer–Lambert equation: I=IoBe−(μρ) xm where $I_{o}$ and I are quantities describing the photons before and after transmission. $\mu_{m}$ is called the mass attenuation coefficient, μρ; a parameter that measures the proportion of the beam which transmits through the barrier without interaction. Another parameter that serves similar purpose (for a medium of mass density ρ) is the linear attenuation coefficient, LAC (μ=μρ∗ρ). In the Beer–Lambert relation, the parameter B is referred to as the photon buildup factor. B accounts for the measure of secondary photons created within and transmitted as the incident photons interact with the attenuating barrier. Other parameters for describing and comparing the level of photon attenuation (shielding) include effective atomic number (Z_eff_), half value thickness (HVT) and mean free path (MFP). 

The level of attenuation of fast neutron and charged particles such as electrons by an interacting medium is often measured by the fast neutron removal cross section and stopping power/continuous slowing down approximation (CSDA) range, CR. 

The photon and fast neutron shielding parameters of PbB30–50 glass systems were computed with the aid of a free, reliable and accurate web-based phy-X/PSD platform [12]. The photon parameters were estimated for photon energies of selected radioactive sources (22, 35, 47, 88, 99, 161, 284, and 364 keV). To get the exposure buildup factor (EBF), the GP interpolation formula was used to get the EBF at the specified energies. The EBF of the glasses were first estimated at standard energies via phy-X/PSD. The five GP parameters of each glass at the source energies of interest were interpolated using the equation: $C=\frac{C_{1}\left(\log E_{2}-\log E\right)+{\;C}_{2}\left(\log E\;-\log E_{1}\right)}{\log E_{2\;}-\log E_{1}}$ where C_1_ and C_2_ are the values of each of the G-P fitting parameters obtained from phy-X/PSD at tabulated energies E_1_ and E_2_ respectively between which E, the energy of interest falls. The obtained fitting parameters were subsequently used to compute EBF based on the well-known GP fitting method [30,31]. On the other hand, the electron SP and CR were evaluated via the ESTAR platform.

## 3. Results and Discussion

### 3.1. Mechanical Properties

**Figure 1:**
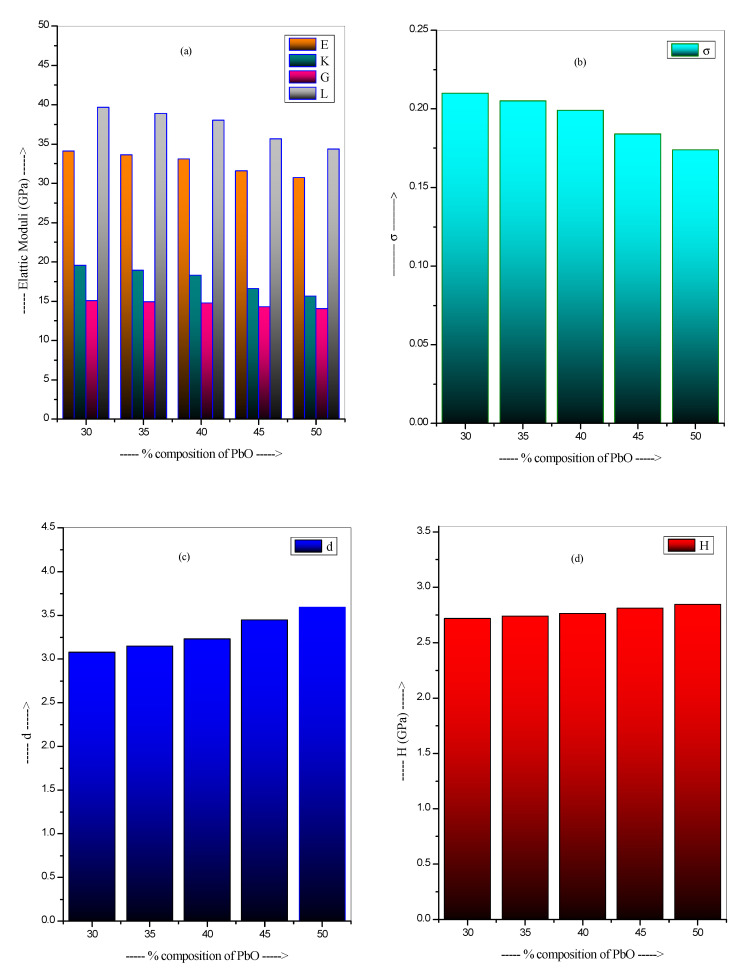
The variation of mechanical properties with the concentration of PbO. (**a**) elastic moduli (**b**) σ (**c**) d (**d**) H.

Mechanical characteristics, as determined by Makishima and Mackenzie (MM), are shown in Table 2. In Figure 1, one can see the variation of these parameters with percentage composition of PbO. The cross-linked glass network’s bond connectivity and stability may be inferred from the values of n_b_. The n_b_ increases from 9.40 × 10^22^ cm^−3^ to 10.09 × 10^22^ cm^−3^. With a greater number of non-bridging oxygens present in the network, there is likely to be a rise in the valence of n_b_, which may imply an increase in the cross-linking of the network. The n_c_ rises from 2.000 to 2.615. For E, the moduli values fall from 34.10 to 30.72 GPa. For K, the moduli values drop from 19.57 to 15.64 GPa. For G, the moduli values drop from 15.08 to 14.04 GPa, and for L, the moduli values fall from 39.67 to 34.36 GPa. As rigidity and mechanical strength diminish with increasing PbO, so does the value of these moduli. The crosslink density of the glasses is defined by the σ. The σ falls between 0.174 and 0.210. The d parameter, which quantifies the degree of cross-linking in the glass network, is crucial. For the current glasses, the approximate value of d is 3.08 to 3.59. This shows that the glasses in use today have 3D networks. With rise in PbO content, the H of the current glasses rising from 2.719–2.847 GPa indicates the increase in connectivity of the selected glasses. 

**Figure 2:**
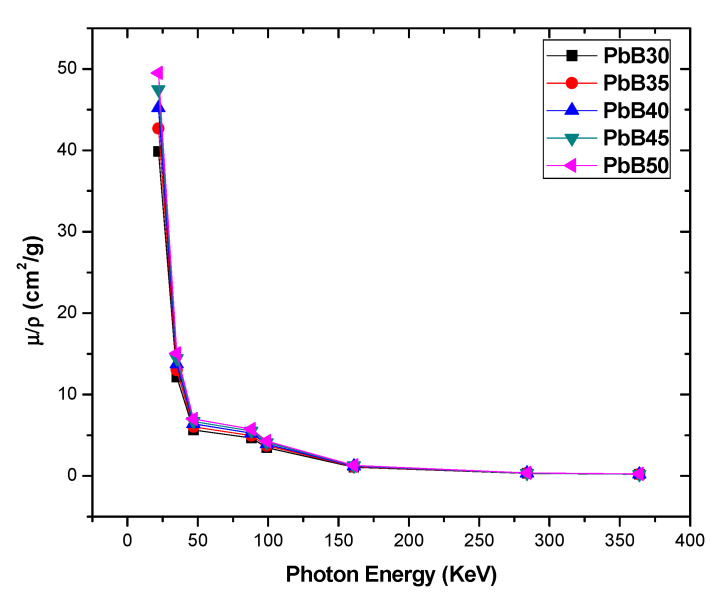
The mass attenuation coefficient of the investigated glass samples.

Figure 2 shows the influence of the photon energy on the computed μρ values of the studied glasses. The figure shows a consistent decay in the value of μρ as energy progresses with the decay appearing to be swift for energies below 88 keV compared to the rest of the energy spectrum. Such smooth decay in μρ indicates relative reduction in the photon absorbing capacity of the glasses at higher energies. This is due to the reduction in the photon interaction cross section of the glasses as energy increases. Within the selected photon energies, two types of photon interactions are generally responsible for the observed behavior of the μρ with energy; these are: photoelectric absorption (PE) and incoherent scattering (InCS) of photons. The cross section for μρ due to PE, (μρ)PE∝E−3 while that due to InCS, (μρ)InCS∝E−1. The (μρ)PE has the dominant effect on μρ at energies (E) in the lower arm of the considered energy spectrum i.e., E<88 keV, hence the observed quick fall in the values of μρ at these energies may be attributed to this, since the values of μρ diminish dramatically at these energies compared to the energy range E > 88 keV where the contribution of (μρ)InCS to μρ becomes more significant. The combined effect due to the energy dependence of (μρ)PE and (μρ)InCS ensures that photon attenuating competence of the glasses decline at higher energies. Hence, μρ peaks at 22 keV with corresponding value of 39.86, 42.68, 45.20, 47.45 and 49.50 cm^2^/g for PbB30–50, while at the end of the energy spectrum, μρ decreased to circa 0.56, 0.49, 0.48, 0.47 and 0.46% of the peak values, respectively. Additionally, conspicuous in Figure 2 is the fact that the differences between the mass attenuation coefficients of the glasses is clearer in the PE dominated (low energy) compared to the InCS dominated (E > 88 keV) region of the energy spectrum. As the influence of InCS becomes significant, the differences in the mass attenuation coefficient of the glasses appears to fade out. At each energy, the differences in the values of μρ follow the trend (μρ)PbB30 < (μρ)PbB35 < (μρ)PbB40 < (μρ)PbB45 < (μρ)PbB50. This trend and the variation in the magnitude of the differences between μρ of the glasses at different energies are enforced by the dependence of both PE and InCS cross sections on the chemical composition of the glasses. For compounds and mixtures, the Z_eff_ is a number which can be used to approximately describe their chemical compositions. The Z_eff_ is usually higher for substances containing a greater proportion of higher atomic number atoms [32]. Thus, it is expected that the Z_eff_ of PbB50 > PbB45 > PbB40 > PbB35 > PbB30 due to the increasing Pb (Z = 82) atomic content by weight as given in Table 2. Furthermore, the fact that (μρ)PE∝Z3 and (μρ)InCS∝Z0 explains the distinct and not so conspicuous differences in μρ values for the glasses in the low and high end of the energy spectrum, respectively. The observed behavior of the μρ of the PbB-glasses with respect to energy and Pb content of the glasses has also been reported in recent times for other Pb-based glass systems [33,34,35,36].

**Figure 3:**
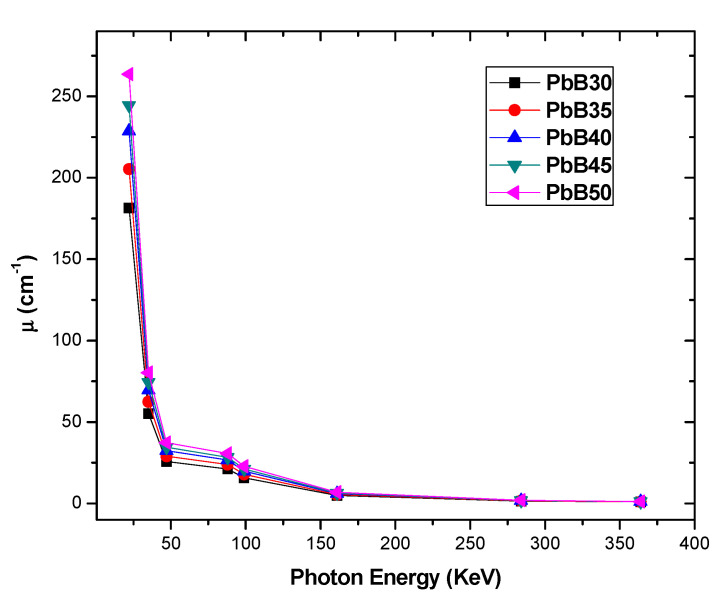
Linear attenuation coefficient µ spectra of PbB30–50 glasses.

The variation of μρ with respect to energy and chemical content is similar to that of µ (Figure 3) due to similar reasons. However, the differences in the µ value of the glasses are greater at similar energy compared to μρ due to the sensitivity of µ to density unlike μρ which has been normalized for density differences. The linear attenuation coefficient is a measure of the relative photon absorption capacity of the glasses at equal thickness (in cm). µ of PbB30–50 glasses decrease from 181.31–0.92, 205.28–1.01, 228.74–1.09, 244.37–1.15 and 263.33–1.21 cm^−1^ as E increases from 22–364 keV. Similar to μρ, the relative close proximity of the values of µ at E E≥ 161 keV is a further attestation that µ is less sensitive to variation in the chemical composition of the glasses at these energies. 

**Figure 4:**
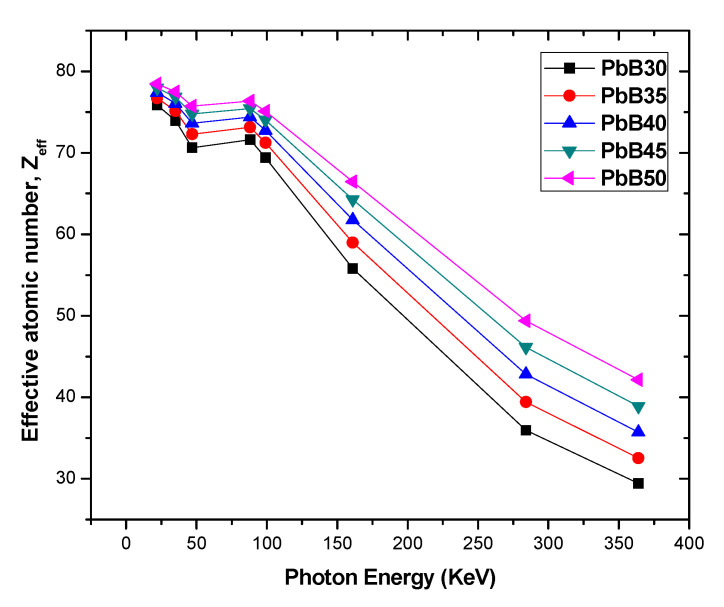
Variation of Z_eff_ with photon energy for PbB30–PbB50 glasses.

The chemical composition effect of an attenuating medium on its photon attenuation capacity is often expressed via the Z_eff_. Z_eff_ depends on photon energy. Variation in the value of Z_eff_ as a function of energy is illustrated in Figure 4. As expected, Z_eff_ declines as energy appreciates due to the dependence of PE and InCS on chemical definition of the glasses and photon energy. Z_eff_ value varies from 29.41 to 78.45 for all the glasses and for all considered photon energies. Figure 4 also showed that glasses with higher Pb atomic content records higher value of Z_eff_, implying that low Z_eff_ indicates low photon shielding capacity and vice versa. 

**Figure 5:**
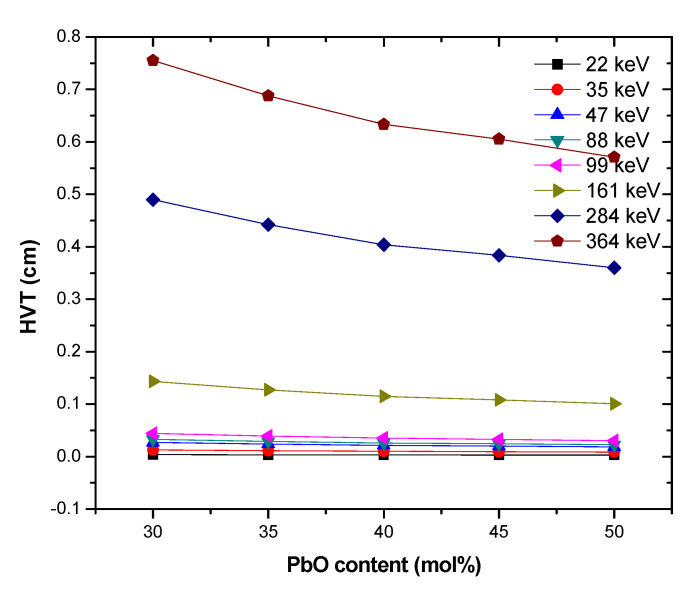
HVT of the investigated glasses at different energies and PbO molar concentration.

The thickness of an attenuating medium required to attenuate incident photons by 50% is termed the half value layer/thickness (HVT). It is an easy parameter for stating and analyzing the shielding prowess of any medium. The effect of PbO molar content of the PbB-glasses on the HVL at different energies is depicted in Figure 5. The figure obviously showed that PbO content of the glasses influences their respective HVT at each photon energy. The growth of PbO molar concentration in the glass system decreases the HVT of the glasses at each energy. The reduction of the HVT is due to the high density and effective atomic number of the glasses with higher PbO content as interaction cross section of photon increases with atomic number and particle density. This is an indication that photon attenuation efficiency is directly proportional to the PbO content in the PbB-glasses. Hence, thicker PbB glasses with lower PbO content is required to achieve similar attenuation level compared to one with higher PbO content and vice versa.

**Figure 6:**
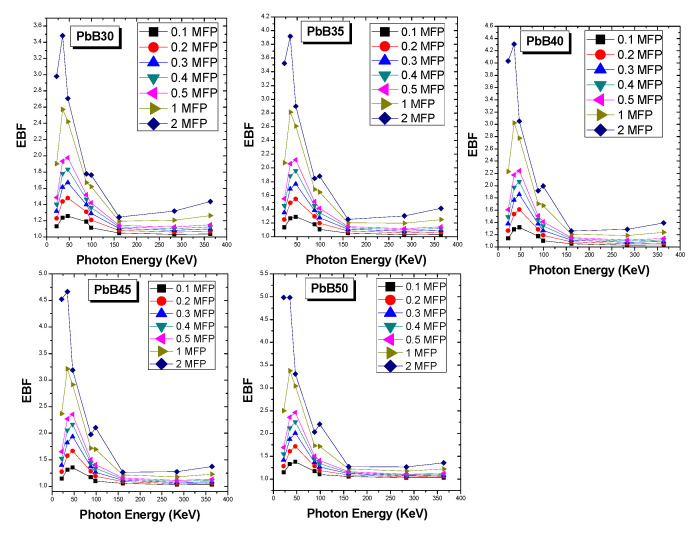
Variation of EBF with energy at selected depth up to 2 MFP.

**Figure 7:**
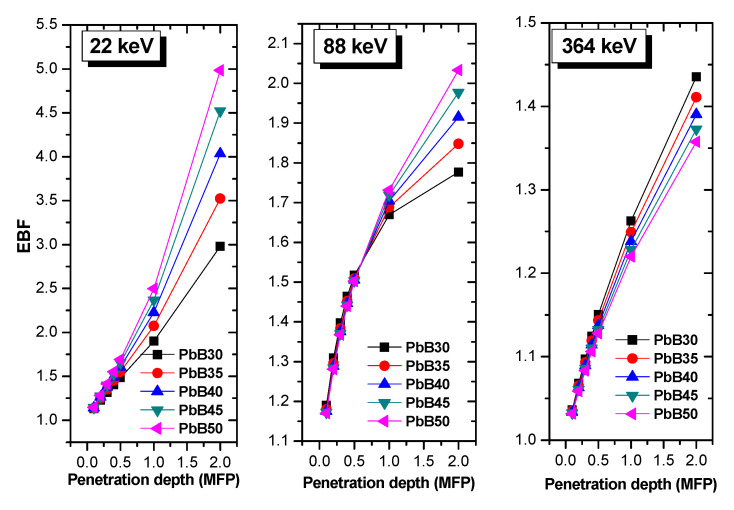
Variation of EBF with penetration depth at selected energies of 22, 88 and 364 keV.

Changes in the EBF of the glasses at selected depths up to 2 MFP at all source energies considered is given in Figure 6. The EBF varies with energy in a analogous fashion for all the glasses. High EBF was recorded at energies corresponding to absorption edges of the atomic species present in the glasses. EBF grows with thickness of the glasses due to multiple scattering at greater depth. Comparing EBF of the glasses at 22, 88 and 364 keV as shown in Figure 7 reveals that EBF of the glasses was directly proportional to the effective atomic number of the glasses at 22 and 88 keV while the reverse is the case at 364 keV. The EBF at the two former energies is due to secondary photons produced by photon electrons produced by the PE process. These electrons are directly proportional to effective atomic number, hence the observed trend. On the contrary, EBF at 364 keV is due to those produced by the InCS process and since the cross section is proportional to $\frac{Z}{A}$; this quantity is inversely proportional to Z_eff_, this explains the reversal of the EBF trend at this energy. Obviously, the shielding efficiency of the PbB-glass shield can be improved by reducing photon buildup at lower energies if lower glass thickness is adopted for shielding. The increment in the PbO content of the glasses improved the photon protection ability of the investigated glasses. 

**Figure 8:**
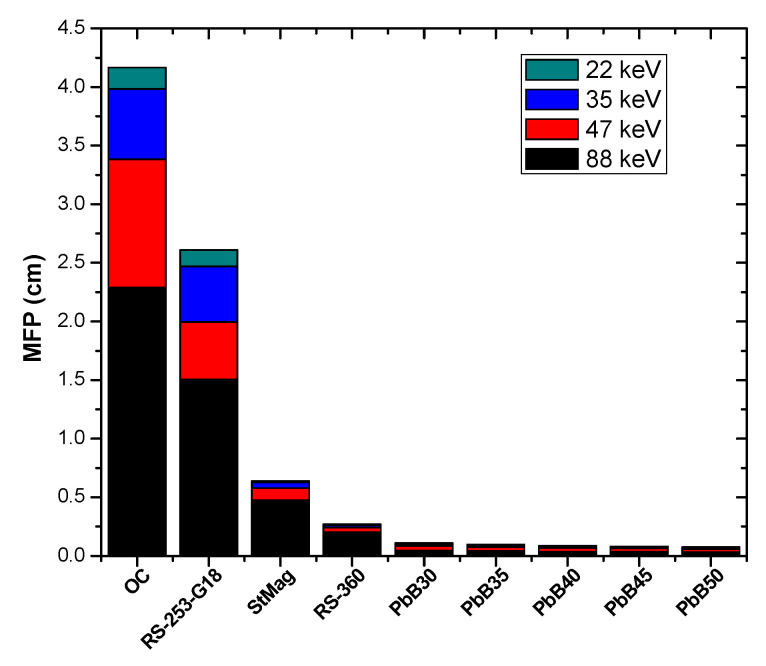
Comparison of MFP value of PbB30–50 glasses with those of some conventional shield at 22, 35, 47 and 88 keV.

**Figure 9:**
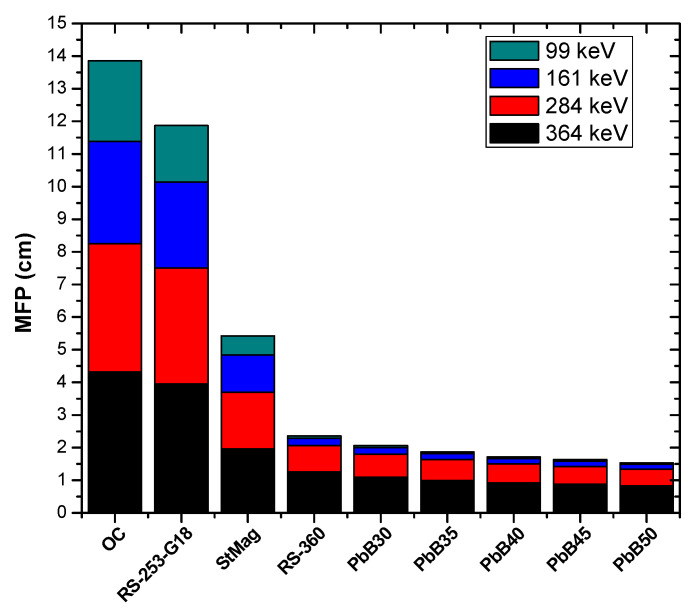
Comparison of MFP value of PbB30–50 glasses with those of some conventional shield at 99, 161, 284 and 364 keV.

In order to compare the shielding power of the investigated glass system with those of conventional shields (ordinary concrete (OC), high density steel magnetite concrete (StMag) and two commercial glass shields containing lead known with the tradenames RS-360 and RS-253-G18), the mean free path (MFP) was used. As a shielding parameter, high MFP indicates that photon moves a large distance before interaction, and hence attenuation is reduced. Figure 8 and Figure 9 show the comparison in the value of MFP of the glass systems and those of other materials (OC, StMag), RS-360 and RS-253-G18) at all the considered photon energies. The figures reveal that MFP increases with energy for all media due to low interaction cross section of photons at higher energies. However, at equal energy, the photon shielding capacity of the PbB-glasses was superior to those of the compared materials. This is a strong empirical indication that the glasses included in the present study possess better photon protection capacity compared to these conventional shields.

### 3.2. Fast Neutron Attenuation Feature

**Figure 10:**
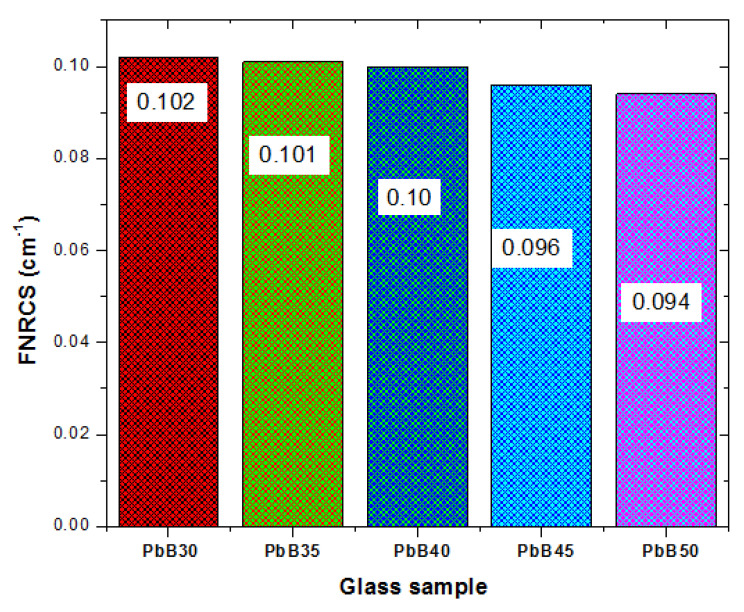
FNRCS of PbB30–PbB50 glasses.

The fast neutron removal cross section (FNRCS) is to fast neutrons what the µ is to photons. The FNRCS (in cm^−1^) is the probability that a fissile/fast neutron will be removed from a fast a neutron beam after first interaction with an attenuating medium. Figure 10 shows the magnitude of the FNRCS of each of the PbB-glasses. The FNRCS decreases in the glasses as Pb and B content increase and decrease, respectively. This is expected as B is a better fast neutron absorber than Pb. The FNRCS value of the glasses varies from 0.094–0.102 cm^−1^. Comparing FNRCS values of PbP30–PbB50 with those of some common fast neutron absorber (such as OC (0.094 cm^−1^), graphite (0.099 cm^−1^) and polyacrylic acid, PPA (0.088 cm^−1^), it is clear that all the PbB glasses are better than PPA while PbB30 is superior to all the compared materials in terms of neutron absorption.

### 3.3. Range and Stopping Power of Electrons

**Figure 11:**
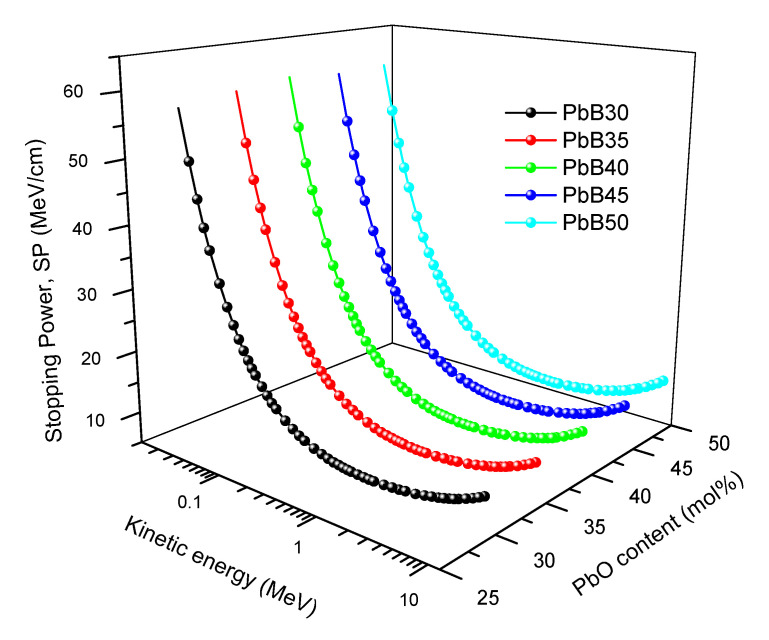
Total SP of electron as function of kinetic energy and PbO content of the PbB-glasses.

**Figure 12:**
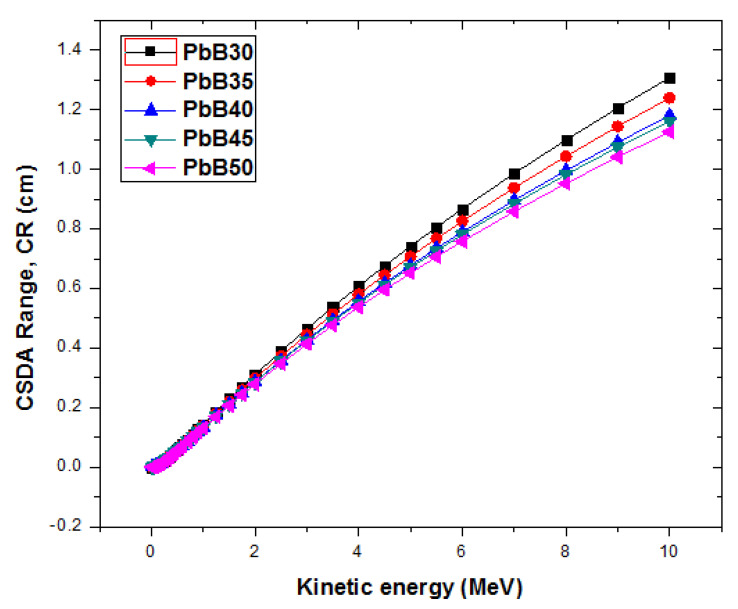
CSDA range of PbB-glasses.

The total stopping powers TSP (MeV/cm) and the continuous slowing down approximation range (CR) of electrons with kinetic energy (T) between 0.01–10 MeV in the glasses were plotted as shown in Figure 11. The movement of electrons through a material results in energy losses due to Coulomb interactions and bremsstrahlung production. The TSP accounts for the energy loss due to these two processes per unit length of the interacting medium. Generally, SP initially decreases with T for T less than 1 MeV for all glasses. This is due to losses resulting from Coulomb interaction whose cross section decreases with energy. At energies greater than 1 MeV, the radiation yield becomes high due to the dominant effect of radiation losses. The increasing radiation yield of electrons with energy ensures that the SP grows with T as observed in Figure 11. Total SP of the glasses increases according to the trend throughout the T spectrum: (SP)_PbB50_ > (SP)_PbP45_> (SP)_PbB40_ > (SP)_PbP35_ > (SP)_PbB30_. Contrary to SP, CR follows a reverse trend. Higher TSP ultimately leads to lower penetration (CR) in the glasses as shown in Figure 12. Figure 11 and Figure 12 show that energetic electrons shielding (like just like photon absorbing) features improve with increase in PbO content of PbB-glasses.

## 4. Conclusions

The Makishima and Mackenzie model has been used to determine the various mechanical properties of the PbO-WO_3_-Na_2_O-MgO-B_2_O_3_ glass system prepared using the melt quenching technique. The values of E, K, G and L moduli decrease from 34.10 to 30.72 GPa for E, 19.57 to 15.64 GPa for K, 15.08 to 14.04 GPa for G and 39.67 to 34.36 GPa for L, respectively, as the PbO increases. Gamma-ray photons, electrons and fast neutron shielding parameters were calculated for the PbB-glasses using standard procedures. Obtained parameters showed that the increase of PbO content of the glasses had strong positive influence on their shielding ability against photons and electrons. Hence, PbB50 had superior photon and charged particle (electron) absorption competence among the PbB glasses. Analysis of fast neutron removal cross sections of the glasses indicated that FNRCS increased/decreased with B_2_O_3_/PbO molar content, respectively, with PbP30 having the highest FNRCS value. The PbB glass sample with the best fast neutron shielding competence possesses the least photon and charged particle absorbing efficacy. Comparing the radiation shielding ability of the glasses with those of existing shielding materials, it is obvious that the investigated glasses have huge potential in radiation protection applications.

## Figures and Tables

**Table 1:** Chemical composition of the selected samples.

Sample	Mole Fraction					Wt. Fraction of Elements Present in the Sample						Density (g/cm^3^)	Molar Volume (cm^3^)
	PbO	WO_3_	Na_2_O	MgO	B_2_O_3_	B	O	Na	Mg	W	Pb		
PbB30	30	10	10	10	40	0.067	0.249	0.036	0.019	0.143	0.485	4.549	28.187
PbB35	35	10	10	10	35	0.056	0.224	0.034	0.018	0.135	0.534	4.81	28.254
PbB40	40	10	10	10	30	0.045	0.200	0.032	0.017	0.128	0.577	5.061	28.369
PbB45	45	10	10	10	25	0.036	0.179	0.030	0.016	0.122	0.616	5.149	29.376
PbB50	50	10	10	10	20	0.027	0.161	0.029	0.015	0.116	0.652	5.326	29.840

**Table 2:** Mechanical properties of the examined samples.

Sample	Mechanical Properties								
	n_b_ (× 10^22^ cm^−3^)	$\bar{n_{c}}$	E (GPa)	K (GPa)	G (GPa)	L (GPa)	σ	d	H (GPa)
PbB30	9.40	2.000	34.10	19.57	15.08	39.67	0.210	3.08	2.719
PbB35	9.70	2.138	33.63	18.96	14.94	38.88	0.205	3.15	2.741
PbB40	9.98	2.285	33.10	18.30	14.78	38.01	0.199	3.23	2.765
PbB45	9.94	2.444	31.59	16.60	14.31	35.67	0.184	3.45	2.812
PbB50	10.09	2.615	30.72	15.64	14.04	34.36	0.174	3.59	2.847

## Data Availability

The data presented in this study are available on request from the corresponding author.
